# Decoding F508del Misfolding in Cystic Fibrosis

**DOI:** 10.3390/biom4020498

**Published:** 2014-05-06

**Authors:** Xiaodong Robert Wang, Chenglong Li

**Affiliations:** 1 Department of Pharmaceutical, Social and Administrative Sciences, McWhorter School of Pharmacy, Samford University, 800 Lakeshore Drive, Birmingham, AL 35229, USA; 2 Division of Medicinal Chemistry and Pharmacognosy, College of Pharmacy, The Ohio State University, Columbus, OH 43210, USA; E-Mail: li.728@osu.edu

**Keywords:** ABC transporter, CFTR, cystic fibrosis, drug discovery, F508del, molecular dynamics simulation, nucleotide-binding domain, protein conformation, protein folding, protein stability

## Abstract

The functional deficiency of the cystic fibrosis transmembrane conductance regulator (CFTR), a plasma membrane chloride channel, leads to the development of cystic fibrosis. The deletion of a phenylalanine at residue 508 (F508del) is the most common cause of CFTR misfolding leading to the disease. The F508del misfolding originates in the first nucleotide-binding domain (NBD1), which induces a global conformational change in CFTR through NBD1’s interactions with other domains. Such global misfolding produces a mutant chloride channel that is impaired in exocytic trafficking, peripheral stability, and channel gating. The nature and atomic details of F508del misfolding have been subject to extensive research during the past decade. Current data support a central role for NBD1 in F508del misfolding and rescue. Many *cis*-acting NBD1 second-site mutations rescue F508del misfolding in the context of full-length CFTR. While some of these mutations appear to specifically counteract the F508del-induced misfolding, others release certain inherent conformational constraints of the human wild-type CFTR. Several small-molecule correctors were recently found to act on key interdomain interfaces of F508del CFTR. Potential rational approaches have been proposed in an attempt to develop highly effective small molecule modulators that improve the cell surface functional expression of F508del CFTR.

## 1. Introduction

Cystic fibrosis is caused by the functional deficiency of a cAMP-activated plasma membrane chloride channel known as the cystic fibrosis transmembrane conductance regulator (CFTR) [1]. Over 90% of the patients carry at least one copy of the CFTR gene, where the deletion of a phenylalanine has occurred at residue 508 (F508del). The F508del mutation causes impaired exocytic trafficking of the nascent CFTR as evidenced by its misprocessing [2]. The F508del CFTR, instead of functioning as a regulated chloride channel on the cell surface, is retained in the endoplasmic reticulum (ER) and subject to proteasome-mediated degradation [3,4].

Interestingly, a dramatic increase in the cAMP-stimulated chloride channel activity was observed by patch clamp analysis in F508del-expressing 3T3 fibroblasts after incubation at 30 °C for 2 days [5]. The increased chloride channel activity was accompanied by improved processing of the mutant protein, suggesting that extended period of incubation at reduced temperature rescues the F508del processing defect, leading to improved cell surface expression of the mutant protein [5]. Nevertheless, the opening probability of the mutant CFTR remained low even after the low temperature incubation [5], indicating that this particular channel gating defect is not fully corrected by the low temperature incubation.

Despite the impaired maturation/export/processing of nascent F508del CFTR, a small fraction of the fully synthesized mutant protein are able to reach the plasma membrane but display reduced stability at the cell periphery [6]. This defect was later found to contribute significantly to the low steady state level of F508del mutant on the plasma membrane [7]. In fact, F508del CFTR molecules rescued from the ER retention by low temperature or certain second-site mutations display reduced stability at the cell periphery at 37 °C [6,8]. Recently, accumulating evidence suggests a major defect in cytoskeletal anchorage of F508del CFTR at the apical plasma membrane [9,10,11,12,13].

The above three-fold defect of F508del CFTR brings significant challenges as well as opportunities to the ongoing drug discovery effort aimed at improving cell surface functional expression of this mutant. A thorough understanding of the various aspects of F508del misfolding will accelerate the development of therapeutics that treat the majority of the patients with this devastating disease.

## 2. NBD1 Misfolding to Domain-Domain Miscontact

**Figure 1:**
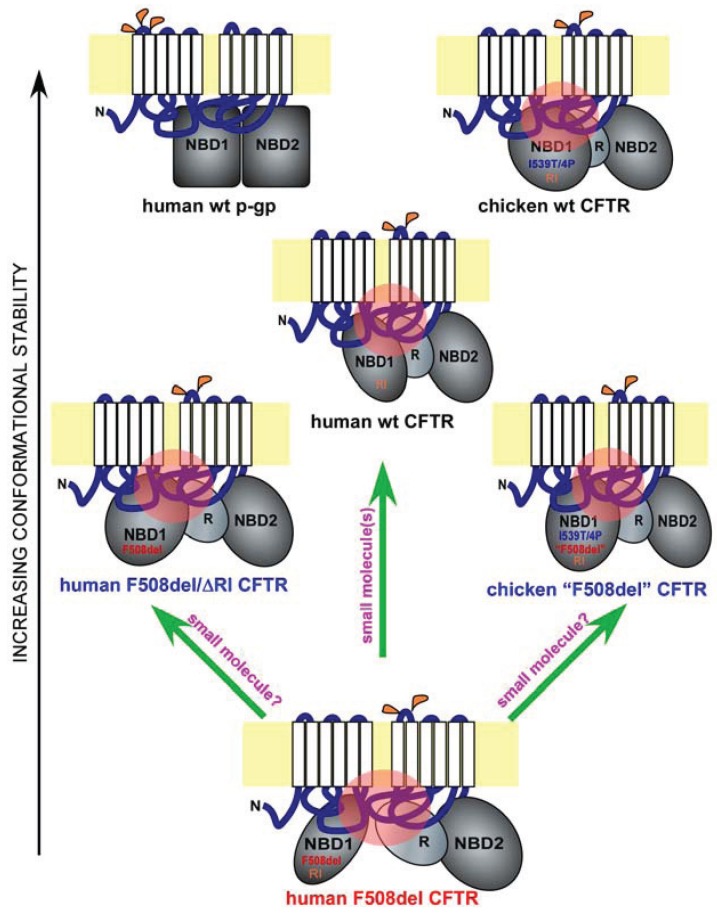
Misfolding and rescue of F508del cystic fibrosis transmembrane conductance regulator (CFTR). Shown are domain conformation of human full-length wild-type (wt) CFTR, its paralogue p-glycoprotein (p-gp), and other CFTR homologues and mutants, as indicated. In each panel, a full-length ABC protein is anchored to the membrane bilayer (in yellow) through two six-pass membrane-spanning domains (white). The extracellular and cytoplasmic loops are in blue, and the major cytoplasmic domains such as the two nucleotide-binding domains (NBD1 and NBD2) and the regulatory domain (R) are in grey. Glycosylation sites on the extracellular loops are labeled in orange (p-gp has three and CFTR has two). The different shapes and orientations of the major cytoplasmic domains represent their different conformations. Key sequence variations in NBD1 are labeled. I539T/4P refers to the combined I539T and four proline substitutions occurring in chicken CFTR. “F508del” refers to the chicken version of the human F508del mutation. RI refers to the presence of the regulatory insertion. Key interdomain contacts for CFTR are labeled by pink circles. The proteins are arranged in the order of conformational stability. Green arrows represent potential F508del rescue strategies.

CFTR is composed of two six-pass membrane-spanning domains (MSDs), each followed by a nucleotide binding domain (NBD). The above two modules are connected by a regulatory (R) domain (Figure 1, human wt CFTR). The F508 residue is located in the NBD1. Early circular dichroism analysis suggested that the deletion of F508 results in a reduced level of ordered secondary structure in an NBD1 peptide in solution [14]. This was the first piece of *in vitro* evidence pointing to the possibility that F508 deletion can alter NBD1 local conformation.

Surprisingly, about twelve years later, the crystal structure of F508del NBD1 containing a number of solubilization mutations revealed no major conformational change as compared with its wild-type counterpart, except for some changes in local surface topography in the vicinity of the deleted F508 residue [15]. This finding is consistent with three-dimensional modeling of the NBD heterodimer, which predicted that the F508 residue is not located in the active site of NBD1 but rather is exposed at the domain surface and hence might play a role in interaction with the MSD [15,16,17]. Consistent with this prediction, *in situ* limited proteolysis of membrane-bound full-length F508del CFTR revealed a more prominent defect in domain-domain interaction than in NBD1 conformation [18]. Additional support came from the structural analysis of F508 substitution mutants, which showed that substitutions that cause conformational change in isolated NBD1 *in vitro* are rather rare, whereas F508 substitutions that cause misprocessing of full-length CFTR are far more common [19]. Together, these data imply that NBD1’s interactions with other CFTR domains might play a critical role in F508del misfolding. Notably, the F508 residue mediates the contact between NBD1 and the intracellular loop 4 (ICL4) residing in MSD2 as demonstrated by molecular modeling [20,21,22] and chemical crosslinking [22] (Figure 1, human wt and F508del CFTR; refer to the pink circles for key interdomain contacts).

## 3. NBD1 Revisited

While the role of F508del mutation in CFTR domain-domain miscontact has been clearly established, it remains unclear as to whether the same mutation produces a conformational change in NBD1. The finding that the F508del NBD1 used for structural analysis [15] contains several solubilization mutations, which not only increase its solubility but also partially rescue its misfolding [23], prompted a revisit of the subject. Using *in situ* limited proteolysis, a quantifiable conformational change in NBD1 was observed in membrane-bound, full-length F508del CFTR [8]. This NBD1 conformational defect is fully correctable by revertant mutation R555K [24] or by low-temperature incubation in permissive cell lines, which is accompanied by conformational rescue in other CFTR domains [8].

Isolated human F508del NBD1, in the absence of additional mutation, has low solubility and is prone to aggregation. This poses a major challenge to biophysical and structural analyses of the domain *in vitro*. This challenge, however, can be circumvented by comparing the biophysical properties of paired wild-type and F508del NBD1s in the presence of the same combinations of solubilization mutations (*i.e.*, sequence matching). One of the combinations used is the simultaneous deletion of two short amino acid sequences known as the regulatory insertion (RI) (Figure 1, human wt CFTR, and chicken wt and “F508del” CFTR) and regulatory extension (RE) [25,26], both of which were found to be disordered and dispensable for CFTR gating [27,28].

Thermal unfolding of sequence-matched wild-type and F508del NBD1s in the presence of various solubilization mutations or their combinations was measured using differential scanning calorimetry [29]. In all the sequence-matched pairs studied, F508del mutation lowers the melting temperature (T_m_) of human NBD1 by 6–7 °C, suggesting that F508 deletion causes thermodynamic destabilization of the isolated NBD1. MgATP increases the T_m_ of both wild-type and F508del NBD1s in a concentration-dependent and saturable manner, suggesting a critical role for nucleotide binding in the conformational stabilization of NBD1.

Isothermal denaturation by increasing concentrations of urea was also performed on isolated NBD1 lacking both RI and RE [30]. Biophysical analyses such as simultaneous circular dichroism, intrinsic fluorescence, and static light-scattering revealed that NBD1 unfolds through two sequential conformational steps: (1) partial unfolding leading to a conformational intermediate that is prone to aggregation; and (2) subsequent complete unfolding. Interestingly, the first step of the unfolding is strongly enhanced by the deletion of F508, and this effect is offset by the presence of MgATP or by second-site revertant mutations. In contrast, the second step is hardly affected by the F508del mutation. Although neither the thermal unfolding nor the isothermal denaturation discussed above faithfully reproduces the misfolding of F508del NBD1 *in vivo*, these data provide important insights into the inherent biophysical impact of F508 deletion on NBD1 peptide conformation.

Hydrogen/deuterium exchange mass spectrometry analysis on matched wild-type and F508del NBD1 constructs with fewer solubilization mutations revealed that F508 deletion increases local backbone dynamics in the vicinity of residues 509–511 [28]. Additional structural analysis of a large set of human F508del NBD1 constructs in the presence of solubilization mutations revealed a consistent conformational defect in NBD1—the solvent exposure of V510, a hydrophobic residue that is normally embedded in the interior of NBD1 [28]. It is highly likely that the aberrant movement of V510 to the surface of NBD1 is the primary driving force for the misfolding of F508del NBD1 and hence for the global conformational change in full-length F508del CFTR. Interestingly, an overlay of two recently published human NBD1 structures [28], a wild-type NBD1 with seven solubilization mutations and an F508del NBD1 with two selected solubilization mutations, revealed the dislocation of the 573–580 loop adjacent to the NBD1 signature motif [31]. The implication of this conformational change is currently unclear. It is possible that such a conformational change might contribute to the reduced opening probability of F508del CFTR. On the other hand, as the two NBD1 constructs are not paired, we cannot rule out the possibility that such a conformational change is caused, at least in part, by the difference in solubilization mutations.

While homology-based static modeling of NBD1 as well as the full-length CFTR protein has provided valuable insights into the potential role of F508 deletion in the global misfolding of CFTR [16,17,20,21,22,31], molecular dynamics simulation has a unique role in revealing the potential folding pathways of F508del NBD1. Regular isothermal–isobaric molecular dynamics simulation of paired wild-type and F508del NBD1s in the presence of the same seven solubilization mutations revealed less stable and more mobile conformational trajectories for F508del NBD1 [32]. We conducted replica-exchange molecular dynamics simulation of human wild-type and F508del NBD1s in the absence of any solubilization mutation [33]. Our simulation, with enhanced sampling, allowed us to compare the unfolding of the “intact” wild-type and F508del NBD1s. We have observed a well defined conformational defect in a fraction of NBD1 conformers harboring the F508del mutation. This conformational defect is characterized by the solvent exposure of V510 and the dislocation of the 509–511 loop [33]. These findings are consistent with the biophysical analyses of NBD1s in the presence of solubilization mutations [28]. More interestingly, reducing the temperature led to a major decrease in the percentage of the defective conformers, consistent with an effect of low temperature on the stabilization of F508del NBD1, favoring wild-type-like conformation [33]. It remains unclear how such NBD1 conformational defects transform into the global misfolding of the full-length CFTR and its subsequent misprocessing. Nevertheless, a recent study on mechanism-based corrector combination alluded to the possibility that NBD1 conformational correction might be the missing ingredient to effective F508del correction [34].

## 4. Regulatory Insertion

CFTR is an atypical member of the ATP-binding cassette (ABC) family of transporters, where it is the only ion channel [1]. Other members of the ABC family include the p-glycoprotein (p-gp), an ATP-dependent efflux pump that transports drugs and other molecules out of the cell [35] (Figure 1, human wt p-gp and wt CFTR). Interestingly, the nascent wild-type p-gp is processed much more efficiently than wild-type CFTR, and the maturation of the former is far less dependent upon major cytoplasmic chaperone Hsp90, suggesting that p-gp has a much higher conformational stability than CFTR [36] (Figure 1, black arrow on the left). One unique feature of the CFTR NBD1 is the presence of a 32-residue peptide sequence between the first two β-strands, which is known as the RI (regulatory insertion) [26] (Figure 1, human wt and F508del CFTR, and chicken wt and “F508del” CFTR). While the RI appears to be dispensable for the channel regulation of human wild-type CFTR by ATP or protein kinase A in *Xenopus* oocytes [27], its deletion rescues the F508del defects in processing, peripheral stability, and channel gating [37]. Apparently, the presence of the RI within CFTR NBD1 increases its propensity to misfolding when F508del is deleted (Figure 1, human wt, F508del, and F508del/ΔRI CFTR). Structural analysis [26], NMR studies [38], and molecular dynamics simulation [37] of NBD1 suggest that the RI region is highly dynamic in both wild-type and F508del NBD1. The removal of the RI might have altered the conformational dynamics of one or more key regions of F508del NBD1 in a manner that improves both the NBD1 conformation and its interactions with other domains, leading to simultaneous improvement in processing, peripheral stability, and channel gating. Future work will reveal the atomic details of the rescue of F508del misfolding by RI deletion.

## 5. A Lesson from Avians

F508del mutation causes CFTR misprocessing in multiple mammalian species. However, the severity of F508del misprocessing and hence the deficiency in its cell surface functional expression are much greater in human as compared with in pig and mouse [39]. This trend continues further in certain non-mammalian species such as avians [40]. Chicken F509del CFTR (corresponding to human F508del CFTR, and denoted as chicken “F508del” CFTR), when expressed in HEK-293 cells, behaves similar to human wild-type CFTR in processing, peripheral stability, and channel gating [40]. The higher body temperature in avians (39–43 °C) might necessitate a higher thermodynamic stability for CFTR. This is largely accomplished by the I539T substitution and four additional proline substitutions at residues 422, 434, 492, and 534 as the same substitutions made in human F508del CFTR restore its processing, peripheral stability, and channel gating [40]. Moreover, reversion of the chicken “F508del” CFTR back to the human F508del-like sequence reproduces the three-fold defect seen in the human F508del mutant [40].

Importantly, all the above residues are located within NBD1. Molecular dynamics simulation revealed that, in the presence of I539T, proline substitutions stabilize the structurally diverse regions of human F508del NBD1 [40]. Such stabilizing effects not only improve channel gating but also restore the interdomain contact between NBD1 and the ICL4 (Figure 1, human wt and F508del CFTR and chicken wt and “F508del” CFTR; refer to the black arrow on the left for relative conformational stability and to the pink circles for key interdomain contacts). These findings are highly consistent with a pivotal role for NBD1 in the F508del-induced defects in channel gating and global conformation. They also provide compelling evidence supporting the notion that NBD1 is a key target for F508del correction.

## 6. Implications for Drug Discovery

Ivacaftor has been approved by the U.S. Food and Drug Administration (FDA) for treatment of 4%–5% of cystic fibrosis patients that carry the G551D substitution mutation [41]. In February, this drug was further approved by the FDA for treatment of patients carrying any one of the eight additional mutations causing CFTR gating defect [42]. However, a drug that treats the over 90% of the patients that carry the F508del mutation remains unavailable. Overcoming F508del misfolding represents the major challenge in turning the latter into reality. Early attempts in this direction have largely relied on high-throughput screening, which yielded a number of F508del correctors [43,44]. However, to date, none of these correctors has risen above the threshold of efficacy that offers significant clinical benefit to cystic fibrosis patients in clinical trials.

While research efforts are still being made using the above approach, scientists in the field are shifting their attention to mechanistic aspects of F508del misfolding and correction. Of particular interest, correctors that target different sites within CFTR are additive in F508del rescue [31,34]. Certain correctors are additive to specific second-site revertant mutations within NBD1 or to low temperature [31]. These findings suggest the existence of multiple intramolecular targets within CFTR for F508del correction, and perhaps more than one small molecules are needed in order to provide sufficient level of correction. It is hoped that elements of rational design will bridge the current technical gap and lead to the successful development of the first FDA-approved F508del corrector or corrector combination for clinical use.

Based on current data, the deletion of F508 induces a global misfolding in CFTR [18], resulting in impaired maturation of nascent channel protein, its subsequent degradation in the ER, and the loss of function at the cell surface [2,3,4]. Nevertheless, even though the mutant protein were able to escape the ER quality control and reach the cell surface, it has a much reduced half-life [6] and is aberrant in channel gating [45]. Therefore, approaches that focus primarily on bypassing the ER quality control will need to be coupled with those that fix the F508del defects at the cell periphery in order to be effective. A viable alternative, however, is to correct the core defect—F508del misfolding, which will hopefully restore the CFTR native conformation and hence simultaneously improve its processing, stability, and channel gating.

Consistent with recent findings, NBD1 is the origin of F508del global misfolding [8,22,28,29,30,33,37,46,47,48,49,50,51]. The current question is whether it is possible to restore F508del NBD1 to both its native conformation and its proper contacts with other key domains. Before this question can be satisfactorily addressed, key conformational determinants of F508del NBD1 misfolding need to be identified, and their specific roles in F508del global misfolding have to be defined. While recent work suggested that the combination of multiple agents are likely needed in order to achieve effective correction [34,52,53], it remains to be determined whether a single agent can accomplish the same (Figure 1, upward green arrow).

Another potential approach to the same problem comes from the fact that human CFTR contains certain sequences that hinder its folding even in the absence of F508del mutation [37,40] (Figure 1, wt, F508del, F508del/ΔRI human CFTR, and wt and “F508del” chicken CFTR). Such sequences include the RI and certain amino acid residues within the human NBD1 such as the I539. The presence of these sequences in human CFTR produces conformational constraints in NBD1 and renders CFTR more prone to misfolding as compared with other members of the ABC family or avian CFTR. Removing or altering such sequences can bypass the F508del-induced CFTR misfolding. It is presently unclear whether the same effects are achievable by small molecule compounds (Figure 1, two slanted green arrows). Future research on the atomic details of F508del misfolding, especially within NBD1, and on CFTR-corrector interactions will provide clues.

## 7. Conclusions

A thorough understanding of the atomic details of the F508del-induced CFTR misfolding will provide key information for the rationale design of highly effective F508del correctors or corrector combinations. NBD1 conformational rescue might be an important part of the equation in terms of effective functional rescue of F508del CFTR.
